# Early Identification of Atherosclerosis in People Living with HIV by Coronary Computed Tomography Angiography

**DOI:** 10.3390/diagnostics16060893

**Published:** 2026-03-18

**Authors:** Müge Toygar Deniz, Özgür Çakır, Burak Acar, Cemile Çakmak, Sibel Balcı, Sıla Akhan

**Affiliations:** 1 Department of Infectious Diseases and Clinical Microbiology, Faculty of Medicine, Kocaeli University, Kocaeli 41001, Turkey; 2 Department of Radiology, Kocaeli University, Kocaeli 41001, Turkey; cakirozgur@hotmail.com; 3 Department of Cardiology, Kocaeli University, Kocaeli 41001, Turkey; burakacarmd@yahoo.com; 4 Department of Biostatistics and Medical Informatics, Kocaeli University, Kocaeli 41001, Turkey

**Keywords:** HIV, atherosclerosis, cardiovascular disease, cardiac imaging techniques

## Abstract

**Background:** The advancements in antiretroviral treatment (ART) have led to a 69% reduction in AIDS-related deaths. However, people living with HIV (PLWH) face age-related comorbitidies like coronary artery disease (CAD), which can be 50% higher compared to HIV-negative individuals. This study explores the prevalence and extent of early CAD in PLWH without a history of cardiovascular disease using computed tomography angiography (CTA). **Methods:** A 320-detector row CTA (Aquilion ONE, Canon Medical Systems) was utilized to determine prevalence of coronary atherosclerosis. Logistic regression analysis and ROC analysis were performed to predict risk factors for the presence of atherosclerosis. **Results:** A total of 186 individuals participated in this study, including 74 PLWH and 112 HIV-seronegative controls. A notable disparity in the occurrence of coronary atherosclerosis was observed between the two groups, with 20% of individuals in PLWH showing plaque in the coronary arteries as detected by CTA, compared to 7% in the control group (*p* = 0.015). In the plaque group, a significant increase in age was observed (*p* = 0.001) along with elevated levels of fasting blood glucose and hemoglobin A1c (*p* < 0.001 and *p* = 0.017 respectively). HIV seropositivity and age were significantly associated with the presence of plaque (aOR, 5.5 [95% CI, 1.7–25.8] and 21.7 [95% CI, 5.5–88] respectively). When evaluating age, fasting blood sugar and HbA1c through ROC analysis to predict plaque presence, age is the strongest predictor, with an AUC of 0.899 (*p* < 0.001, 95% CI: 0.847–0.939) and a cutoff value of 35 years. Additionally, HbA1c and fasting blood sugar had an AUC of 0.664 (*p* = 0.0047, 95% CI: 0.574–0.746) and 0.759 (*p* < 0.001, 95% CI: 0.688–0.822) respectively. Youden cutoff values were 5.5 for HbA1c and 92.4 for fasting blood sugar. **Conclusions:** The higher prevalence of CAD in PLWH may indicate that inflammation is a substantial risk. It is important to remember that CAD can develop early in PLWH. Moreover, including HbA1c and fasting blood sugar measurements in routine follow-up may help facilitate earlier detection of atherosclerosis.

## 1. Introduction

The use of more potent antiretroviral treatment (ART) regimens and increased access to treatment among people living with HIV (PLWH) have resulted in increased life expectancy. According to UNAIDS data, a 69% reduction in AIDS-related deaths has occurred since its peak in 2004 [1]. As they age, these patients are at an increased risk of age-related comorbidities such as cardiovascular disease (CVD), cancer, liver, and renal disease.

PLWH have an increased risk of CVD, which can be 50% or higher [2]. Compared to HIV-negative individuals, the relative risk of CVD incidence in PLWH is generally 1.5- to 2-fold greater [3]. Furthermore, the rates of myocardial infarction, heart failure, stroke, and various other CVD manifestations, including pulmonary hypertension and sudden cardiac death, are significantly higher in PLWH, even in the setting of viral suppression with effective ART [4]. Therefore, accurately assessing an individual’s risk of CVD is significant for implementing necessary preventive measures [5].

The risk factors of coronary artery disease (CAD) among PLWH include not only traditional factors but also chronic HIV infection itself, persistent low-grade inflammation, and the cardiometabolic effects associated with ART [6,7]. Proteins associated with HIV such as Tat, Nef, and gp120, play significant roles in the onset and acceleration of atherosclerosis [8]. Also, traditional risk factors such as age, gender and race contribute to atherosclerosis by promoting endothelial dysfunction and inflammation. Moreover, metabolic impairments, such as dyslipidemia and insulin resistance, are prevalent in PLWH [9].

Advanced imaging techniques, such as coronary computed tomography angiography (CTA), can elucidate the processes of CAD by offering a comprehensive analysis of heart tissue, assessing the structure of coronary arteries, and detecting myocardial or vascular inflammation [10]. In the literature, it has been suggested that CTA is useful for the early identification of subclinical atherosclerosis in PLWH, particularly for detecting non-calcified plaques that are vulnerable to rupture. In addition, it is possible to determine Agatston calcium scores, calcium volumes and coronary artery calcium scoring (CACS) using CTA. These scores have been found to be a good indicator of HIV-associated atherosclerosis [11]. The main objective of this study was to evaluate subclinical aterosclerosis in PLWH who were asymptomatic and had no prior history of cardiovascular disease.

## 2. Material and Methods

### 2.1. Study Design

This retrospective cohort study aimed to compare the prevalence of CAD in asymptomatic PLWH to that of an HIV-seronegative control group (CG). The primary endpoint of this study was the prevalence of coronary plaque. Secondary endpoints included the degree of coronary stenosis, Agatston calcium score, calcium volume score, and cardiovascular risk assessment, all evaluated using CTA.

### 2.2. Study Population

This study included one hundred and eighty-six people. Seventy-four PLWH and 112 HIV-negative CG were recruited for this study. Participants were aged ≥ 18 years and had no known cardiac disease or symptoms suggestive of cardiac disease (including angina, arrhythmia, valvular heart disease, pericarditis, congestive heart failure, or any prior treatment for coronary artery disease or heart disease). PLWH who had been receiving ART for more than six months were included in the study. Patients with inaccessible data, as well as those with a history of coronary disease, were excluded. Eligible patients were called by telephone and their additional diseases and comorbidities were inquired. When patients were contacted by telephone to verify their comorbid conditions, they were also specifically questioned to ensure the absence of active cardiac symptoms. Participants underwent CTA for screening purposes due to the presence of comorbidities or cardiovascular risk factors. CTA images obtained between 2020 and 2025 using the same device were retrieved from the Picture Archiving and Communication Systems (PACS) and interpreted by a single radiologist. Demographic and laboratory parameters were obtained from the hospital’s data system.

### 2.3. Definitions

Hypertension was defined as blood pressure ≥ 140/90 mm Hg and/or current use of antihypertensive treatment [12]. Dyslipidemia was defined as low-density lipoprotein cholesterol ≥ 3.0 mM (116 mg/dL) and/or current use of lipid-lowering agents as previously described [13]. Diabetes mellitus was defined as nonfasting plasma glucose ≥ 11.1 mmol/L (≥200 mg/dL) and/or HbA1c ≥ 6.5%and/or use of antidiabetic agents [14].

### 2.4. Cardiac Computed Tomography Angiography

**Figure 1:**
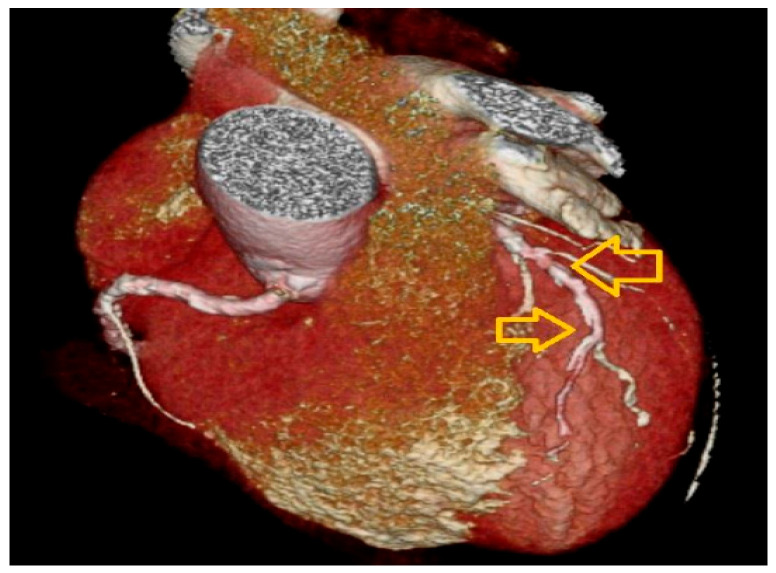
Three-dimensional (3D) volume-rendered (VR) image of the heart. This reconstruction holistically visualizes the anatomical course of the coronary arteries on the heart, its surface and the extensive calcifications (bright white structures). The yellow arrows draw attention to the widespread calcified plaques on the left anterior descending (LAD) artery. This case represents a moderate calcium burden consistent with the cohort median values.

**Figure 2:**
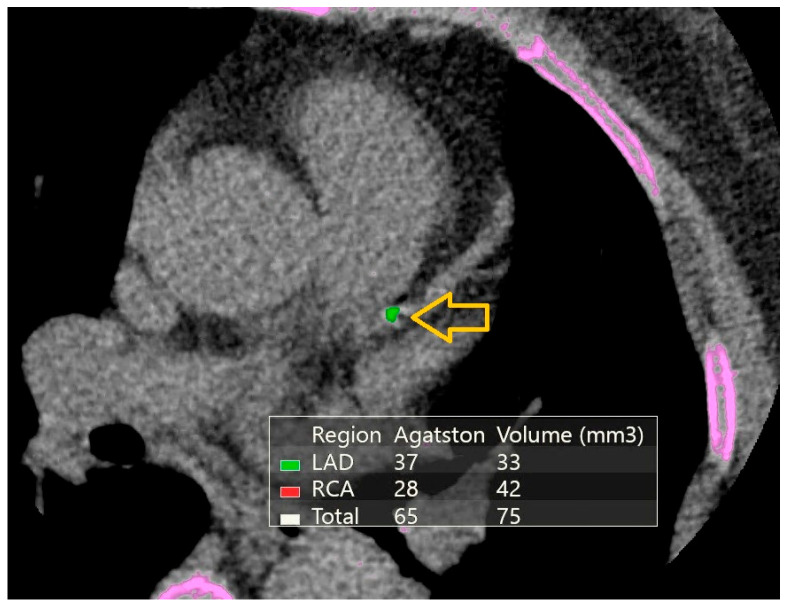
Axial non-contrast cardiac CT image with calcium scoring overlay. Focal calcified plaques are visible within the left anterior descending (LAD) and right coronary artery (RCA) (yellow arrows). The embedded table summarizes the Agatston calcium scores and calcium volumes: LAD:37 (33 mm^3^), RCA:28 (42 mm^3^), total Agatston score 65 and total calcium volume 75 mm^3^. This case illustrates a moderate coronary calcium burden representative of the study population. The pink area represents bone tissue.

A 320-detector row CTA (Aquilion ONE, Canon Medical Systems, Otawara, Tochigi, Japan) was utilized (Figure 1). Before undergoing the scan, participants with a heart rate exceeding 60 beats per minute were administered an intravenous β-blocker (metoprolol 5–20 mg), provided there were no contraindications to β-blockers and their systolic blood pressure was above 100 mmHg. Image capture took place during breath-held inspiration. Following the standard procedure, a test bolus of 20 mL contrast agent was injected at a rate of 5 mL/s to establish the best timing for contrast injection. Contrast agent (80–100 mL, iopamidol, Isovue; Bracco Diagnostics, Inc., Princeton, NJ, USA) was injected intravenously at a rate of 5 mL/s to ensure homogeneous enhancement of the entire coronary artery tree. Axial images were reconstructed with a slice thickness of 0.5 mm and increment of 0.4 mm using a half-scan algorithm with a temporal resolution of 165 ms. All reconstructions were transferred to an offline workstation for analysis (Vitrea 2, version 3.3, Vital Images) (Figure 2).

**Figure 3:**
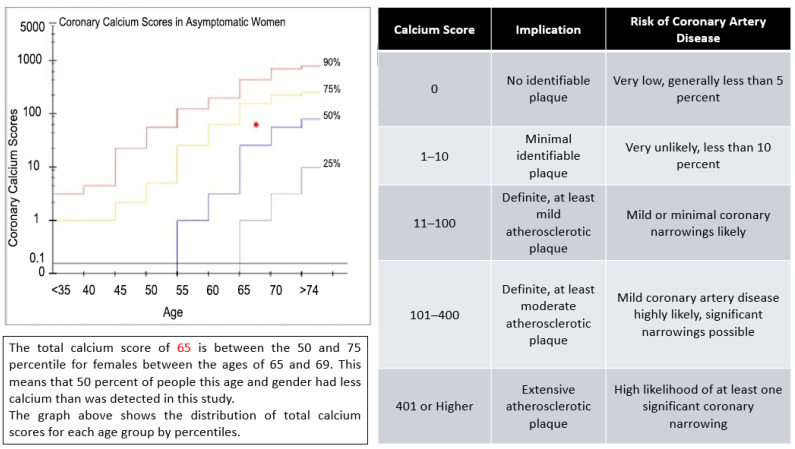
Calcium percentile chart and age–sex-adjusted coronary artery calcium (CAC) percentile distribution. Reference percentile curves demonstrate the distribution of coronary calcium scores across age groups in asymptomatic women. The highlighted data point corresponds to a total Agatston score of 65 in a 65–69-year-old female, located between the 50th and 75th percentile. This indicates an intermediate age-adjusted calcium burden relative to the reference population.

Plaque presence, including both calcified and non-calcified coronary atherosclerotic plaques, was assessed by a single experienced radiologist blinded to participants’ clinical history and HIV status. Severe stenosis was defined as >70% luminal narrowing in any segment. Figure 3 presents calcium scores and volumes using the Agatston method [15], along with age-based coronary artery calcium scores (CACS).

### 2.5. Ethical Approval

The study was conducted in accordance with the Declaration of Helsinki and was approved by the Ethics Committee of Kocaeli University (no: KÜ GOKAEK-2025/10/04, Project Number: 2025/164). All the participants provided informed consent for participation.

### 2.6. Statistical Analysis

Statistical evaluation was performed using IBM SPSS (version 29.0; IBM Corp., Armonk, NY, USA). Compliance with a normal distribution will be evaluated using the Kolmogorov–Smirnov and Shapiro–Wilk tests. Variables showing normal distribution are given as mean ± standard deviation, and variables not showing normal distribution are given as median (25th–75th percentile). Categorical variables were presented as frequencies (percentages). Differences between groups will be determined with an independent sample *t*-test for variables with normal distribution and with the Mann–Whitney U test for variables without a normal distribution. Relationships between numerical variables were evaluated using Pearson’s or Spearman’s correlation analysis. Relationships between categorical variables were determined using chi-square analysis. A logistic regression model was used in multivariate analyses. In hypothesis tests, *p* ≤ 0.05. was considered sufficient for statistical significance.

## 3. Results

A total of 186 individuals participated in this study, including 74 sex- and age-matched PLWH and 112 HIV-seronegative CG. Demographic and clinical characteristics of individuals are shown in Table 1. PLWH had a known HIV infection duration with a median of 3 years (IQR 2–7). All PLWH were undergoing ART. Of these, 50 (67.6%) were administered a regimen consisting of tenofovir alafenamide, emtricitabine, and bictegravir. In addition, 15 individuals (20.3%) received a combination of dolutegravir and lamivudine. Six individuals (8.1%) were treated with tenofovir disoproxil, emtricitabine, and dolutegravir, whereas three individuals (4.1%) were treated with tenofovir alafenamide, emtricitabine, cobicistat, and elvitegravir. In general, the immunological response was well-maintained, with a median CD4 cell count of 684 cells/μL (IQR 484–904) and a CD4/CD8 ratio of 0.9 (IQR 0.7–1.4). Additionally, 56 individuals (75.7%) had an undetectable viral load. There were no significant differences between the groups in traditional cardiovascular risk factors such as hypertension, diabetes, and dyslipidemia (all *p* > 0.05). Among metabolic parameters, only HDL levels were lower in the PLWH group (*p* = 0.03).

Furthermore, median C-reactive protein (CRP) levels were significantly higher in the PLWH group (*p* = 0.025). Similarly, IL-6 levels were significantly elevated in PLWH compared with controls (*p* = 0.003).

A notable disparity of coronary atherosclerosis was observed between the two groups, with 20% of individuals in PLWH showing plaque in the coronary arteries as detected by CTA, compared to 7% in the control group (*p* = 0.015). Table 2 presents a comparison of all CTA-derived parameters among the study participants. In the CACS risk assessment, incorporating CAC, patients were classified into the following risk categories: 0–25 as very low, 25–50 as low, 50–75 as moderate, 75–90 as high, and 90–100 as very high risk. In CG, the distribution of patients with plaques was as follows: two individuals were classified as very low risk, five individuals were classified as low risk, and one individual was classified as moderate risk. In contrast, among PLWH, the plaque distribution revealed a broader spectrum of risk categories: one individual was classified as very low risk, one individual as low risk, six individuals as moderate risk, five individuals as high risk, and one individual as very high risk. Although there was no statistically significant difference according to risk assessment because of the sample size, PLWH are concentrated at high risk levels. In the present study, plaque characterization (calcified, non-calcified, and mixed) was not systematically categorized; therefore, subgroup analysis according to plaque composition was not performed.

**Figure 4:**
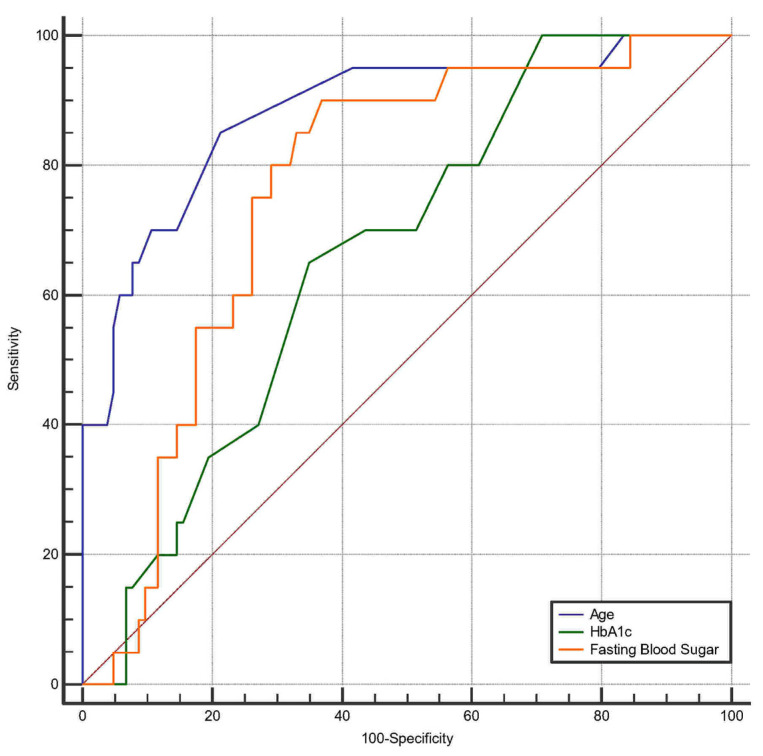
Receiver operating characteristic curves for age, HbA1c and fasting blood sugar to classify patients with plaque.

Table 3 presents a comparison of the significant parameters between the groups with and without plaque among all participants. Age was significantly higher in the plaque group (*p* < 0.001). In the plaque group elevated levels of fasting blood glucose and hemoglobin A1c were observed (*p* < 0.001 and *p* = 0.017 respectively). The increase in total cholesterol and the decrease in the CD4/CD8 ratio approached statistical significance in the plaque group. In the multivariate regression analyses, age and HIV seropositivity were significantly associated with the presence of plaque (Table 4). (aOR for plaque presence, 21.7 [95% CI, 5.3–88] and 5.5 [95% CI, 1.17–25.8], respectively). When evaluating age, fasting blood sugar and HbA1c through ROC analysis to predict plaque presence, age is the strongest predictor, with an AUC of 0.899 (*p* < 0.001, 95% CI: 0.847–0.939) (Figure 4). Additionally, HbA1c and fasting blood sugar had an AUC of 0.664 (*p* = 0.0047, 95% CI: 0.574–0.746) and 0.759 (*p* < 0.001, 95% CI: 0.688–0.822) respectively. The Youden cutoff values were determined to be 35 for age, 5.5% for HbA1c, 92.4 for fasting blood sugar, in relation to plaque presence.

## 4. Discussion

Our findings demonstrated that subclinical atherosclerosis was significantly more frequent in PLWH (20%) compared with sex- and age-matched HIV-negative individuals (7%). Consistent with our results, in the Swiss HIV cohort, the prevalence estimates for any coronary atherosclerosis in PLWH 16% [16]. Zhou et al. observed a prevalence of 44.3% in PLWH and 32.9% in HIV-negative controls [17]. The higher prevalence observed in this study may be attributable to the older median age of the participants and the substantially higher rate of dyslipidemia (58%) in the PLWH group. There were no significant differences between the groups in traditional cardiovascular risk factors such as hypertension, diabetes, and dyslipidemia in our study. Knudsen et al. recently demonstrated that PLWH exhibited a twofold increase in the odds of any coronary atherosclerosis and a threefold increase in the odds of obstructive coronary atherosclerosis in subjects with similar traditional cardiovascular risk factors [18]. Moreover, in a study conducted by Ferrer et al., who assessed subclinical atherosclerosis in the carotid and iliofemoral territories, the prevalence was reported to be 40% [19]. However, this relatively high rate may be attributable to the use of ultrasound as the imaging modality.

HIV infection is associated with elevated levels of multiple pro-inflammatory molecules, including IL-6 and CRP [20]. A recent review also reported increased plasma levels of chronic inflammatory biomarkers in PLWH, including IL-6 (1.16–5-fold increase) and CRP (1.32-fold increase) [21]. Consistent with previous reports, our study also demonstrated higher CRP and IL-6 levels in PLWH. Chronic inflammation in PLWH, reflected by elevated IL-6 and CRP levels, contributes to endothelial dysfunction and the development of atherosclerosis [22]. However, in our study, CRP levels did not differ significantly between patients with and without plaque. In addition, IL-6 levels could not be compared between the groups due to the limited sample size. Therefore, larger studies with adequate power are needed to better elucidate the potential contribution of HIV-associated inflammation to the development of atherosclerosis.

Previous studies have demonstrated CAD can be associated with CAC scores, which can be determined via CTA [23,24]. In our study, Agatston calcium scores and calcium volume were lower in PLWH compared with the HIV-negative group; however, this difference did not reach statistical significance. In PLWH, CAC scores may underestimate coronary atherosclerotic burden, because non-calcified plaques which are more prevalent in this population are not detected by CAC scoring [25]. Consistent with this observation, previous studies have reported a higher prevalence of non-calcified coronary plaques in PLWH [26,27]. Although coronary CTA provides the ability to characterize plaque morphology and distinguish between calcified, non-calcified, and mixed plaques, plaque composition was not systematically assessed in the present study. This represents an important limitation, particularly given prior evidence indicating that PLWH tend to exhibit a higher prevalence of non-calcified and potentially vulnerable plaques. The absence of a significant difference in Agatston scores between groups, despite the higher prevalence of plaque among PLWH, may therefore reflect underlying differences in plaque composition that are not adequately captured by calcium-based scoring systems. Future prospective studies incorporating detailed plaque phenotyping are warranted to further elucidate mechanisms underlying HIV-associated coronary atherosclerosis.

In our study, although we did not find a statistically significant difference in age-based CACS because of the sample size, it was observed that PLWH are concentrated in higher risk categories. Recently, a review from the Netherlands highlighted that CACS is particularly valuable in refining cardiovascular risk assessment. Elevated CACS values (>100 or >75th percentile adjusted for age, sex, and ethnicity) can reclassify individuals from intermediate to high risk, thereby identifying those who may benefit from preventive pharmacotherapy [28]. Therefore, we believe that age-based CACS may provide more meaningful risk stratification in PLWH compared with Agatston or calcium volume. However, further large cohort studies are needed to clarify this approach.

In our study, CTA-derived parameters—including left ventricular ejection fraction, end-diastolic volume, end-systolic volume, stroke volume, cardiac output, myocardial mass, and myocardial volume—were numerically lower in PLWH. Notably, end-diastolic volume emerged as the only parameter that differed significantly between the groups, with lower values observed in PLWH. A recent study conducted by Hu et al. left ventricular diastolic function was lower in PLWH than controls [29]. Freiberg et al. showed that PLWH had an overall increased risk of heart failure with preserved ejection fraction (HFpEF) (hazard ratio, 1.21; 95% CI, 1.03–1.41), which may be associated with reduced end-diastolic volume [30]. Similarly, Vasconcellos et al. evaluated 1195 PLWH using echocardiography and concluded that the observed diastolic abnormalities may represent early indicators of future HFpEF development [31].

We found that age, fasting blood sugar, and hemoglobin A1c levels were significantly elevated in individuals with plaque. The literature indicates that elevated fasting glucose levels are associated with persistently high mortality rates due to ischemic heart disease [32]. Recently, a study involving 3312 patients demonstrated that higher HbA1c levels were significantly associated with increased rates of coronary atheroma progression and adverse clinical outcomes [33]. Additionally, the elevation in total cholesterol and the reduction in the CD4/CD8 ratio approached statistical significance in individuals with plaque, indicating the need for careful monitoring. Soto et al. noted that tobacco use, age, and a CD4/CD8 ratio below 0.7 can predict the presence of subclinical atherosclerosis in primary prevention settings. Furthermore, a CD4/CD8 ratio below 0.3 was identified as a diagnostic indicator of atherosclerosis in HIV in the same study [34]. In our study, age and HIV positivity were identified as the independent risk factors for plaque development in the multivariate analysis. When evaluating age using ROC analysis, this emerged as the strongest marker, with an AUC of 0.899 and a cutoff value of 35 years. This suggests that although the EACS guidelines recommend screening individuals aged 40 years and older, atherosclerosis may already be detectable at even younger ages. Nevertheless, it should be acknowledged that the 35-year threshold identified through ROC analysis represents a data-driven observation specific to our cohort and should not be interpreted as a definitive clinical cut-off. Further studies in larger, independent populations are required to validate the robustness and clinical applicability of this finding.

The main limitation of our study is its retrospective design, which limits our ability to determine the causal factors underlying atherosclerosis. In addition, the lack of baseline coronary CTA prior to ART initiation makes it difficult to interpret the progression of atherosclerosis over time. Furthermore, given the limited sample size and the small number of plaque events, only variables that were significant in univariate analyses or considered clinically relevant were included in the multivariate model. This approach may result in wide confidence intervals and carries a potential risk of overfitting, which should be considered when interpreting the findings. Finally, the lack of systematic follow-up data regarding clinical cardiovascular outcomes represents another limitation of our study, as the relationship between subclinical plaques and subsequent ischemic events could not be evaluated. However, compared with previous studies in the literature, the absence of significant differences in traditional cardiovascular risk factors between the groups and the age- and sex-matched design of our cohort substantially strengthens the reliability of our findings.

## 5. Conclusions

In conclusion, identifying subclinical coronary atherosclerosis by using CTA allows us to recognize PLWH who may have an underestimated cardiovascular risk. However, rather than serving as a universal screening modality, coronary CTA may be more appropriately considered as a second-line imaging strategy in individuals with unfavorable metabolic profiles or elevated cardiovascular risk. Moreover, including HbA1c and fasting blood sugar measurements in routine follow-up may help facilitate earlier detection of atherosclerosis. Age and HIV seropositivity emerged as independent predictors of plaque presence, raising the possibility that CAD may develop earlier in PLWH and underscoring the importance of early preventive strategies.

## Figures and Tables

**Table 1:** Demographics and clinical characteristics of individuals.

Characteristics	PLWH (*n* = 74)	Controls (*n* = 112)	*p* Value
Age (years), median (IQR)	35 (31.7–37)	34 (32–35)	0.104 ^a^
Male, *n* (%)	70 (94.6)	97 (86.6)	0.130 ^b^
Smoking, *n* (%)	37 (71.2)	33 (55.9)	0.144 ^b^
Alcohol, *n* (%)	5 (9.6)	5 (8.5)	1 ^b^
Hypertension, *n* (%)	7 (9.6)	12 (13)	0.656 ^b^
Diabetes mellitus, *n* (%)	4 (5.5)	14 (15.1)	0.086 ^b^
Dyslipidemia, *n* (%)	6 (8.2)	8 (8.8)	1 ^b^
**Metabolic parameters, median (IQR)**			
Total cholesterol (mg/dL)	179.5 (143–210)	182 (160–210)	0.357 ^a^
HDL cholesterol (mg/dL)	39.9 (34.9–48.8)	43.4 (36.1–52.3)	**0.030 ^a^**
LDL cholesterol (mg/dL)	103 (84–131.7)	103 (82–133)	0.939 ^a^
Triglycerides (mg/dL)	145 (76.9–194)	128 (76.4–220)	0.926 ^a^
Hemoglobin A1c (%)	5.3 (5.0–5.6)	5.5 (5.1–5.8)	0.067 ^a^
Fasting blood sugar (mg/dL)	90 (83–99)	91.9 (85–100)	0.148 ^a^
BMI (kg/m^2^)	25.1 (21.7–28)	25.8 (23.5–29.4)	0.065 ^a^
**Inflammatory parameters, median (IQR)**			
CRP (mg/L)	6 (3.1–10.5)	2.9 (1–8)	**0.025 ^a^**
IL-6 (pg/mL)	5.1 (4–5.5)	3.1 (2–4.4)	**0.003 ^a^**

**Abbreviations:** PLWH: People living with HIV, IQR: Interquartile range, BMI: Body mass index, LDL: Low-density lipoprotein, HDL: High-density lipoprotein, CRP: C-reactive protein. Boldface *p*-values indicate statistical significance (*p* < 0.05). ^a^ Mann–Whitney U test, ^b^ Chi-square test.

**Table 2:** CTA parameters of all participants.

Parameter	PLWH (*n* = 74)	Controls (*n* = 112)	*p* Value
Presence of coronary plaque, *n* (%)	15 (20.3)	8 (7)	**0.015 ^a^**
Percentage of stenosis, median (IQR)	40 (25–52)	45 (40–60)	0.330 ^b^
Agatston calcium score, median (IQR)	59.5 (33.5–206.2)	87.5 (40–189.5)	1 ^b^
Calcium volume score, median (IQR)	69 (39.7–206.5)	86 (44–200.2)	0.920 ^b^
Coronary stenosis > 50%, *n* (%)	4 (28.6)	4 (50)	0.386 ^a^
LV Ejection fraction, median (IQR)	59.5 (53.7–69.5)	62 (54–67)	0.910 ^b^
End diastolic volume, median (IQR)	130 (115–150)	143 (130–168)	**0.036 ^b^**
End systolic volume, median (IQR)	55 (36.5–70)	56 (38–67)	0.733 ^b^
Stroke volume, median (IQR)	81 (71–94.5)	88 (74–102)	0.317 ^b^
Cardiac output, median (IQR)	5.8 (5.1–6.7)	6.6 (5.4–7.5)	0.079 ^b^
Myocardial mass, median (IQR)	98 (86–114)	104 (81–122)	0.678 ^b^
Myocardial volume, median (IQR)	93.5 (81.7–108)	98 (78–116)	0.538 ^b^
LV/RV regurgitation, median (IQR)	25.5 (13.5–38)	23 (12–33)	0.439 ^b^

**Abbreviations:** PLWH: People living with HIV, IQR: Interquartile range, LV: Left ventricule, RV: Right ventricule. Boldface *p*-values indicate statistical significance (*p* < 0.05). ^a^ Chi-square test ^b^ Mann–Whitney U test.

**Table 3:** Comparison of parameters between participants with and without coronary plaque.

	Plaque Present (*n* = 23)	No Plaque (*n* = 163)	*p* Value
Age (years), median (IQR)	60 (46–74)	34 (32–36)	**0.001 ^a^**
Male, *n* (%)	22 (95.7)	145 (89)	0.476 ^b^
Smoking, *n* (%)	8 (66.7)	62 (62.6)	1 ^b^
Hypertension, *n* (%)	5 (21.7)	14 (9.9)	0.149 ^b^
Diabetes mellitus, *n* (%)	3 (13)	15 (10.5)	0.719 ^b^
Dyslipidemia, *n* (%)	4 (14.7)	10 (7.1)	0.112 ^b^
Hemoglobin A1c (%), median (IQR)	5.6 (5.3–5.9)	5.4 (5–5.7)	**0.017 ^a^**
Fasting blood sugar (mg/dL), median (IQR)	100 (95–127)	90 (83.8–96.9)	**<0.001 ^a^**
Total cholesterol (mg/dL), median (IQR)	195 (174–216)	177 (148–205)	0.080 ^a^
CD4/CD8 ratio, median (IQR)	0.72 (0.3–1.3)	1.08 (0.74–1.5)	0.067 ^a^
CRP (mg/L), median (IQR)	5.2 (1.2–8.4)	4.5 (1–8)	0.816 ^a^

**Abbreviations:** IQR: Interquartile range, CRP: C-reactive protein. Boldface *p*-values indicate statistical significance (*p* < 0.05). ^a^ Mann–Whitney U test ^b^ Chi-square test.

**Table 4:** Logistic regression analyses for plaque presence.

Variable	Univariate		Multivariate	
	OR (95% CI)	*p*	aOR (95% CI)	*p*
HIV seropositivity				
No	-	-	-	-
Yes	3.305 (1.32–8.25)	**0.010**	5.5 (1.17–25.8)	**0.030**
Dyslipidemia				
No	-	-	-	-
Yes	2.75 (0.78–9.6)	0.113	1.08 (0.17–6.8)	0.928
Age				
<35	-	-	-	**-**
≥35	23.59 (8.33–66.76)	**<0.001**	21.7 (5.3–88)	**<0.001**
HbA1c	1.062 (0.77–1.46)	0.714	1.28 (0.73–2.23)	0.385
Fasting blood sugar	1.008 (0.99–1.01)	0.126	1 (0.98–1.02)	0.900
HDL	1.02 (0.99–1.05)	0.057	1.02 (0.98–1.06)	0.200

**Abbreviations:** OR: Odds ratio, aOR: Adjusted odds ratio, CI: Confidence interval, HIV: Human immundeficiency virus, HDL: High-density lipoprotein, HbA1c: Hemoglobin A1c. Boldface *p*-values indicate statistical significance (*p* < 0.05).

## Data Availability

The datasets generated during and/or analyzed during the current study are available from the corresponding author on reasonable request.
